# Cortisol Deficiency in Lenvatinib Treatment of Thyroid Cancer: An Underestimated Common Adverse Event

**DOI:** 10.1089/thy.2021.0040

**Published:** 2022-01-17

**Authors:** Salvatore Monti, Federica Presciuttini, Maria Grazia Deiana, Cecilia Motta, Fedra Mori, Valerio Renzelli, Antonio Stigliano, Vincenzo Toscano, Giuseppe Pugliese, Maurizio Poggi

**Affiliations:** Endocrinology and Diabetes Unit, Azienda Ospedaliero-Universitaria Sant'Andrea, “Sapienza” University, Rome, Italy.

**Keywords:** fatigue, lenvatinib, primary adrenal insufficiency, thyroid cancer

## Abstract

***Background:*** Lenvatinib treatment has shown a significant improvement in progression-free survival in patients with metastatic, progressive, radioiodine-refractory differentiated thyroid cancer, although its use is associated with considerable toxicity. Fatigue is one of the most frequent adverse events (AEs). It has been reported that adrenal insufficiency (AI) may be involved in lenvatinib-related fatigue. In our study, we assessed the pituitary/adrenal axis before and during treatment, and the possible involvement of AI in lenvatinib-related fatigue. This was done to clarify the incidence, development, and time course of AI during lenvatinib treatment.

***Methods:*** We studied 13 patients who were selected for lenvatinib therapy. Adrenal function was evaluated by measuring cortisol and adrenocorticotropic hormone (ACTH) levels and through the ACTH (250 μg) stimulation test.

***Results:*** During treatment, seven patients (54%) developed AI. High levels of ACTH were observed in accordance with the diagnosis of primary AI (PAI). By evaluating the first ACTH test, before starting lenvatinib treatment, we found that patients with <646.6 nmol/L cortisol peak had an increased risk of developing PAI during lenvatinib treatment. Fatigue was observed in 11 patients (84.6%) during lenvatinib treatment. Cortisone acetate treatment induced an improvement in fatigue in six of seven patients (85.7%) in the PAI group, without the need to change the lenvatinib dosage.

***Conclusions:*** PAI may be considered one of the most common AEs associated with lenvatinib. Our data strongly suggest that PAI could be involved in lenvatinib-associated fatigue, particularly in patients with extreme fatigue. In this context, early diagnosis of PAI is essential, especially since glucocorticoid replacement therapy can induce a significant improvement in fatigue, without the need to reduce the dosage of lenvatinib. However, further studies are required to confirm these preliminary findings.

## Introduction

Lenvatinib is an oral, multitarget tyrosine kinase inhibitor (TKI), approved for the treatment of progressive radioiodine-refractory differentiated thyroid cancer (RR-DTC) (1,2). In the study of (E7080) lenvatinib in differentiated cancer of the thyroid, the median progression-free survival was 18.3 months using lenvatinib, and 3.6 months using placebo (3). However, adverse events (AEs) occurred in almost all patients (97.3%) treated with lenvatinib (3).

Although supportive care may be an option for managing AEs, according to the severity of AEs, lenvatinib must be discontinued, interrupted, or administered at a reduced dosage (3), which decreases treatment efficacy. Fatigue is one of the most frequent AEs that lead to discontinuation of lenvatinib treatment.

In 2017, at our Endocrine Center, a patient with lenvatinib-related fatigue developed primary adrenal insufficiency (PAI); cortisone acetate (CA) therapy improved fatigue in the patient (unpublished observation). In 2019, Colombo *et al.* demonstrated PAI in four of the seven patients affected by RR-DTC during treatment with lenvatinib; CA relieved fatigue in them (4).

PAI is a severe and potentially life-threatening condition (5–7). The majority of PAI symptoms are nonspecific, and diagnosis is frequently delayed (6). The simultaneous presence of undiagnosed PAI in patients with RR-DTC may have a strongly negative impact on the morbidity and mortality of patients treated with lenvatinib.

We evaluated the pituitary/adrenal axis before and during treatment to clarify the incidence, development, and time course of PAI during lenvatinib treatment. In addition, the possible impact of PAI on lenvatinib-related fatigue was evaluated.

## Materials and Methods

We analyzed consecutive patients with RR-DTC who started lenvatinib treatment between June 2017 and November 2019 at our Endocrine Center. All patients showed progression according to RECIST 1.1 criteria (8). Eligible patients received no prior therapy with TKIs.

Patients with a history of adrenal disease, adrenal metastases, nephrotic syndrome, and liver disease, or who had been or were being treated with steroids or any drug known to interfere with steroid hormone secretion and metabolism were considered noneligible.

We only included patients treated with lenvatinib for at least 6 months. Performance status was evaluated for each patient using the Eastern Cooperative Oncology Group (ECOG).

AEs were assessed using the National Cancer Institute Common Terminology Criteria for Adverse Events (CTCAE) version 4.03 (9), and lenvatinib was discontinued, interrupted, or reduced according to the severity of AEs.

As this is a retrospective analysis of de-identified data collected as part of the routine clinical procedure, ethical review and approval was not required, but written informed consent was obtained from all patients. The study was conducted in accordance with the provisions of the Declaration of Helsinki, local legislation, and institutional requirements.

All patients were evaluated before and after starting lenvatinib treatment by performing medical history, physical examination, laboratory analysis, and hormonal evaluation. Patients underwent clinical examinations weekly for the first month, and monthly thereafter. Laboratory analysis was scheduled every month. Adrenal function was evaluated before and during lenvatinib treatment every 3 months by measuring cortisol and adrenocorticotropic hormone (ACTH) levels and using the ACTH (cosyntropin; 250 μg) stimulation test. Peak cortisol levels below 500 nmol/L, after ACTH injection, at 30 or 60 minutes, indicate adrenal insufficiency (AI), according to the most recent recommendations (5).

In patients with AI, CA was started at a dosage of 25–37.5 mg/day, according to the weight and clinical conditions (5). In all these patients, the ACTH-test was repeated after 3 months and, in patients with sufficient follow-up, after 6–9 months, after stopping CA for 48 hours.

All hormones were analyzed using a chemiluminescence immunoassay. The sensitivity of the assays was 1.13 pg/mL for ACTH and 0.5 nmol/L for cortisol; the intra-assay and interassay coefficients of variations were 4.9% and 8.9% for ACTH and 4.3% and 5.5% for cortisol (LIASON XL Analyzer). Normal values were 4.7–48.8 pg/mL for ACTH and 101–536 nmol/L for cortisol.

### Statistical analyses

A descriptive analysis of all the sample parameters was carried out. The normality of the distribution of our continuous quantitative variables was demonstrated through the Shapiro–Wilk test and confirmed with the Kolmogorov–Smirnov and D'Agostino and Pearson tests. Continuous quantitative variables were reported as mean and standard deviation (SD). The qualitative variables were presented as absolute frequencies and percentages.

Statistical differences between the two groups of continuous variables were assessed using paired or unpaired Student *t* test, when appropriate. The comparison of frequencies between the groups of qualitative variables was analyzed using the chi-square or Fisher test, when appropriate. The correlation between different variables was determined using the Spearman test. The cortisol peak performance after the ACTH test was evaluated using receiver operating characteristic (ROC) curve analysis and the most accurate cutoff calculated with sensitivity and specificity. Statistical significance was set at *p* < 0.05, and all tests were two sided. All statistical analyses were performed using GraphPad Prism 8 (Graph Pad Software, Inc.).

## Results

From June 2017 to November 2019, 23 patients with progressive RR-DTC started lenvatinib. Ten patients were excluded from our study (five had a follow-up of less than 6 months; five were taking glucocorticoids for problems related to RR-DTC).

The characteristics of 13 patients enrolled are reported in Tables 1 and 2.

**Table 1.** Clinical and Pathological Features of the Patients Treated with Lenvatinib

Patient No., Sex	Tumor histotype, variant	Metastasis	ECOG status	Age at lenvatinib, start (years)	Lenvatinib starting dose (mg)	Follow-up (months)	PAI (yes/no)	PAI diagnosis after lenvatinib started (months)	AEs (Grade)^a^
1, F	PTC, classic	Lung, neck	0	63	14	6	No	/	Hypertension (2), proteinuria (1), fatigue (2), palmar plantar erythrodysesthesia syndrome (1), arthralgia/myalgia (1)
2, M	Hurthle cell	Lymph nodes, lung	0	44	24	28	No	/	Hypertension (1), proteinuria (1)
3, F	PTC, solid	Lymph nodes, lung	0	40	24	9	No	/	Hypertension (1), proteinuria (1), diarrhea (2)
4, M	FTC	Lung, neck, bone	0	77	20	6	Yes	3	Hypertension (2), proteinuria (1), fatigue (2), diarrhea (2), stomatitis (1), palmar plantar erythrodysesthesia syndrome (1), dysphonia (1)
5, M	FTC	Lymph nodes, lung	1	79	24	29	No	/	Hypertension (2), proteinuria (1), fatigue (3), diarrhea (2), weight loss (3) stomatitis (1), nausea/vomiting (1)
6, F	PTC, classic	Lung, bone	1	75	14	24	Yes	3	Hypertension (2), proteinuria (2), fatigue (3), weight loss (2), stomatitis (1), nausea/vomiting (1), dysphonia (1)
7, F	PTC, classic	Lymph nodes, lung	1	82	10	6	No	/	Hypertension (1), fatigue (1), diarrhea (1), weight loss (1), stomatitis (1)
8, F	PTC, tall cell	Lymph nodes, lung	0	67	24	15	Yes	3	Hypertension (2), fatigue (2), stomatitis (2), palmar plantar erythrodysesthesia syndrome (3)
9, M	PTC, tall cell	Bone, neck	0	73	24	15	Yes	9	Hypertension (2), fatigue (3), diarrhea (2), weight loss (2), stomatitis (1), palmar plantar erythrodysesthesia syndrome (1)
10, F	PTC, classic	Lung, bone	0	61	24	29	Yes	18	Hypertension (2), fatigue (3), diarrhea (1), weight loss (3), stomatitis (1), palmar plantar erythrodysesthesia syndrome (2), nausea/vomiting (1), dysphonia (1), alopecia (1)
11, F	PTC, solid	Lymph nodes, lung, bone	0	68	24	29	Yes	6	Hypertension (2), proteinuria (2), fatigue (2), diarrhea (2), weight loss (2)
12, F	PTC, follicular	Lymph nodes, lung	1	66	24	27	Yes	12	Hypertension (2), proteinuria (1), fatigue (2), weight loss (3)
13, M	PTC, tall cell	Lymph nodes, lung, bone, brain	0	56	20	6	No	/	Hypertension (2), fatigue (2), weight loss (2), nausea/vomiting (1)

Some AEs of lenvatinib treatment may be symptoms related to PAI: fatigue, weight loss, diarrhea, nausea, and vomiting. In our study, only fatigue was associated with PAI (see the Results and Discussion sections); none of the other symptoms was significantly associated with PAI.

^a^ AEs were assessed using the National Cancer Institute CTCAE version 4.03 (9).

AEs, adverse events; CTCAE, Common Terminology Criteria for Adverse Events; ECOG, Eastern Cooperative Oncology Group; F, female; FTC, follicular thyroid cancer; M, male; PAI, primary adrenal insufficiency; PTC, papillary thyroid cancer.

**Table 2.** Baseline Characteristics of Patients Treated with Lenvatinib

Characteristic	All patients	PAI	No-PAI
No.	13	7	6
Age, years, mean ± SD	65.46 ± 12.82	69.57 ± 5.65	60.67 ± 17.45^a^
>60 Years, *n* (%)	10 (77)	7 (100)	3 (50)
≤60 Years, *n* (%)	3 (23)	0 (0)	3 (50)
Sex, *n* (%)			
Female	8 (61.5)	5 (71.4)	3 (50)
Male	5 (38.5)	2 (28.6)	3 (50)
Weight, kg, mean ± SD	70.59 ± 13.64	71 ± 13.2	70.12 ± 15.39^a^
Performance status, *n* (%)			
ECOG 0	9 (69.2)	5 (71.4)	4 (66.7)
ECOG 1	4 (30.8)	2 (28.6)	2 (33.3)
Starting dose lenvima, mg, mean ± SD	20.8 ± 4.93	22 ± 3.83	19.33 ± 6.02^a^
Follow-up, months, mean ± SD	17.62 ± 10.22	20.71 ± 8.84	14 ± 11.3^a^
TSH, μIU/mL, mean ± SD	0.095 ± 0.074	0.078 ± 0.083	0.115 ± 0.065^a^
ACTH, pg/mL, mean ± SD	29.84 ± 9.4	31.9 ± 5.64	27.4 ± 12.7^a^
Cortisol, nmol/L, mean ± SD	384.6 ± 73.86	396.5 ± 60.33	370.7 ± 91.08^a^
Peak cortisol, nmol/L (ACTH test), mean ± SD	644.5 ± 81.53	600.9 ± 36.42	695.4 ± 90.01^b^
>646.6, *n* (%)		1 (14.3)	5 (83.3)
≤646.6, *n* (%)		6 (85.7)	1 (16.7)

^a^ No significant difference with PAI group.

^b^ Significant difference between PAI groups.

ACTH, adrenocorticotropic hormone; no-PAI, without primary adrenal insufficiency; SD, standard deviation; TSH, thyrotropin.

Before and during lenvatinib treatment, all our patients had electrolytes, hemoglobin, albumin, and renal function in the normal range. Moreover, there were no significant alterations in glycemia or hepatic function.

### Primary adrenal insufficiency

**FIG. 1.**
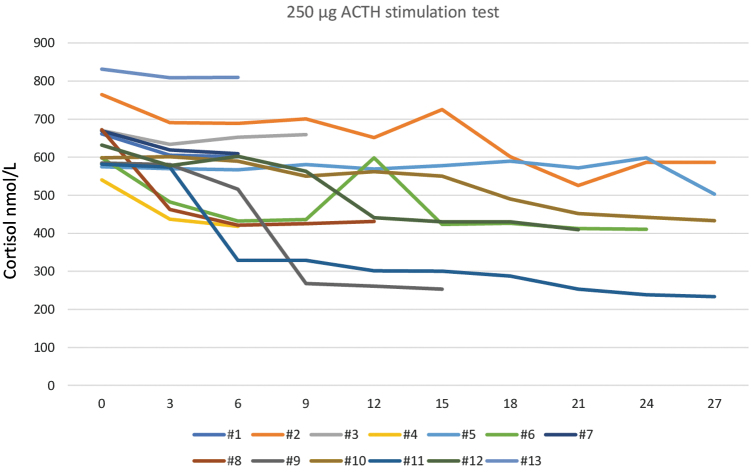
ACTH (250 μg) stimulation test of all 13 patients treated with lenvatinib at baseline (0, before starting therapy) and during follow-up (3, 6, 9, 12, 15, 18, 21, 24, and 27 months). Seven patients (#4, #6, #8, #9, #10, #11, and #12) had an insufficient response (cortisol peak <500 nmol/L) indicating adrenal insufficiency. After 12 months of treatment, PAI patient #6 interrupted lenvatinib for 34 days and ACTH stimulation was normal; after lenvatinib restarted, a recurrence of PAI (cortisol peak <500) was demonstrated (see Results). ACTH, adrenocorticotropic hormone; PAI, primary adrenal insufficiency. Color images are available online.

None of the 13 patients had PAI before starting treatment; in all the cases, the cortisol peak was higher than 500 nmol/L (Fig. 1 and Table 3). During lenvatinib treatment, 7 of the 13 patients (54%, CI 0.2249–0.852) had PAI (Fig. 1); the cortisol peak at PAI diagnosis was 392.7 ± 67.78 nmol/L (mean ± SD). The mean time for development of PAI was 7.71 ± 5.7 months. Six of the seven (86%) patients developed PAI within 12 months of starting lenvatinib treatment (Fig. 1 and Table 3).

**Table 3.** Adrenal Function Before and During Lenvatinib Treatment

Patient No./sex	Cortisol peak before lenvatinib (nmol/L)	ACTH before lenvatinib (pg/mL)	PAI (yes/no)	PAI diagnosis after lenvatinib started (months)	Cortisol peak at PAI diagnosis (nmol/L)	ACTH at PAI diagnosis (pg/mL)
1/F	661	16.2	No	/	/	/
2/M	765	21.1	No	/	/	/
3/F	670	11.9	No	/	/	/
4/M	540	26.7	Yes	3	418	56.8
5/M	575	39.4	No	/	/	/
6/F	598	25.2	Yes	3	432	70.2
7/F	669	34	No	/	/	No
8/F	672	26.4	Yes	3	421	31.5
9/M	584	39	Yes	9	268	95.7
10/F	598	36.5	Yes	18	452	65.3
11/F	582	34.2	Yes	6	328	91
12/F	632	35.3	Yes	12	430	197.1
13/M	832	42	No	/	/	/

ACTH normal level: 6–48.8 pg/mL. None of the 13 patients had PAI before starting treatment; in all cases the cortisol peak was higher than 500 nmol/L. Seven of the 13 patients developed PAI during lenvatinib treatment; cortisol peak after ACTH stimulation and ACTH level were reported at the time of PAI diagnosis in the seven patients. Six of the seven patients with PAI had increased ACTH levels at the time of diagnosis. In one patient without an increase in ACTH levels (patient #8), we excluded a pituitary origin by evaluating other pituitary hormones using pituitary magnetic resonance. However, during follow-up, this patient manifested high levels of ACTH (maximum value 61.5 pg/mL, 9 months after the start of lenvatinib treatment).

Patients with a subnormal response started the CA replacement treatment. The ACTH-test was repeated after 3 months, stopping CA for 48 hours. A further ACTH-test was repeated after 6–9 months in the patients with sufficient follow-up; in all cases AI diagnosis was confirmed.

After 1 year of treatment, lenvatinib was interrupted for 34 days in a 76-year-old patient with PAI (Fig. 1, patient #6) because of surgical complications after cholecystectomy. In this patient, we repeated the ACTH-test after appropriate interruption of CA; the cortisol peak was normal. CA was stopped, and lenvatinib was restarted at the same dosage (14 mg) taken before cholecystectomy. However, after 3 months of lenvatinib treatment, two consecutive ACTH-tests demonstrated recurrence of PAI.

The mean cortisol peak after the first ACTH-test before starting lenvatinib treatment was significantly lower in the PAI group than in the no-PAI group (Table 2).

We assessed the presence of a possible cortisol peak cutoff to predict the development of PAI during lenvatinib. Using the ROC curve analysis, the most accurate cortisol peak threshold was <646.6 nmol/L with 85.71% sensitivity and 83.33% specificity (area under the curve 0.8333, CI 0.5685–1.098, *p* = 0.0452). Using this cutoff, only one of the six patients with cortisol peak >646.6 nmol/L developed PAI, whereas six of the seven patients with <646.6 nmol/L peak developed PAI (*p* = 0.0291; relative risk 5.143; sensitivity 85.71%, specificity 83.33%, positive predictive value 85.71%, and negative predictive value 83.33%).

The cortisol peak in the ACTH-test decreased significantly during lenvatinib treatment.

**FIG. 2.**
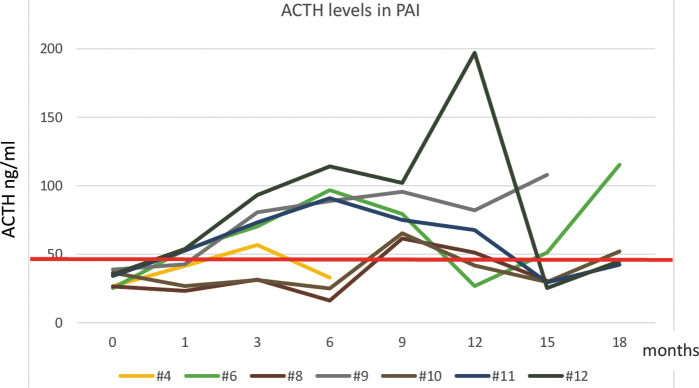
ACTH levels of seven patients that developed PAI, at baseline (0, before starting therapy) and during follow-up, until PAI diagnosis. The red line is the upper limit of the reference range 48.8 pg/mL. In six of seven PAI patients (#4, #6, #9, #10, #11, and #12), ACTH levels increased before diagnosis of PAI. Only #12 had very high ACTH levels (greater than twofold the upper limit of reference range) at the time of PAI diagnosis. In the only patient without an increase in ACTH levels (Fig. 3, patient #8), we excluded pituitary origin by evaluating other pituitary hormones and pituitary magnetic resonance. However, during follow-up, this patient manifested high levels of ACTH (maximum value 61.5 pg/mL). Color images are available online.

Before lenvatinib treatment, ACTH levels were normal in all patients. PAI diagnosis was associated with an increase in the ACTH levels (Fig. 2 and Table 3).

Six of the seven patients (85.6%) with PAI had an increase in ACTH levels, 5 ± 2.68 months before PAI diagnosis. In one patient without an increase in ACTH levels (Fig. 2 and Table 3, patient #8), we excluded a pituitary origin by evaluating other pituitary hormones and pituitary magnetic resonance.

PAI was diagnosed in 7 of the 10 patients aged >60 years and in none of the 3 patients aged ≤60 years (Table 2); however, the difference was not statistically significant (*p* = 0.0699).

The development of PAI was not associated with initial weight, follow-up duration, ECOG status, sex, or starting dose or dose intensity of lenvatinib.

In all the cases enrolled, adrenal metastases or other adrenal gland alterations were excluded by whole-body computed tomography scans and whole-body [^18^F]-fluoro-2-deoxy-D-glucose-positron emission tomography/computed tomography performed for tumor assessment.

### Fatigue

None of our patients complained of fatigue before initiating lenvatinib therapy. During follow-up, fatigue was observed in 11 patients (84.6%, Table 1). The mean time of onset of fatigue was 6.64 ± 6.6 weeks (range 2–23) and the onset of fatigue was early, 2–4 weeks, in most cases (72.7%).

Fatigue was demonstrated in all 10 patients aged >60 years and in 1 of the 3 patients aged ≤60 years (*p* = 0.0330, relative risk 14.67; sensitivity 100%, specificity 91.67%, positive predictive value 66.67%, and negative predictive value 100%).

**FIG. 3.**
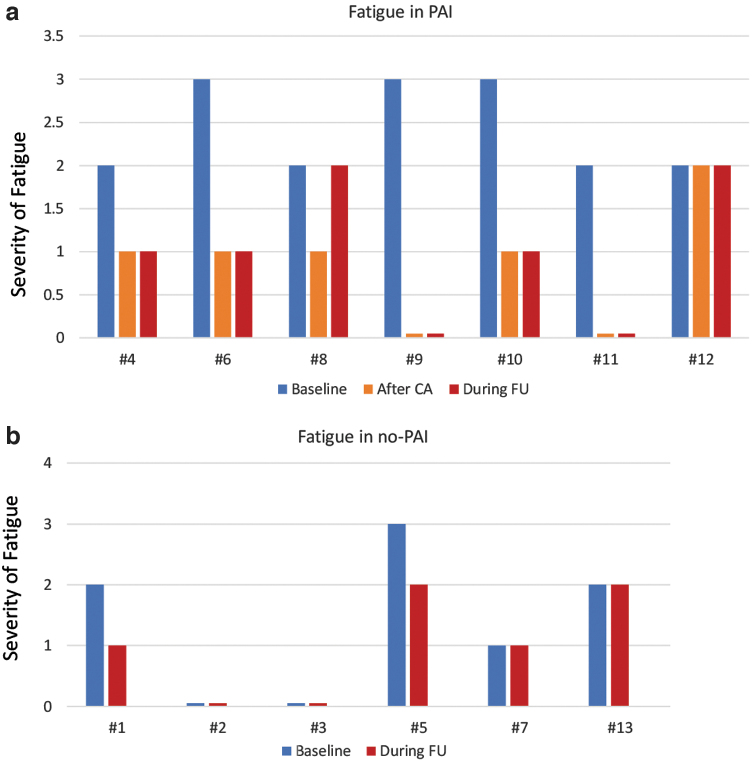
Fatigue in patients with PAI and without PAI (no-PAI). Fatigue was evaluated by the National Cancer Institute CTCAE version 4.03 (9). Fatigue is defined as a disorder characterized by a state of generalized weakness with a pronounced inability to summon sufficient energy to accomplish daily activities. According to CTCAE, three grades of severity of fatigue were identified: Grade 1, fatigue relieved by rest; Grade 2, fatigue not relieved by rest, limiting activities of daily living, such as preparing meals, shopping for groceries or clothes, using the telephone, and managing money; Grade 3, fatigue not relieved by rest, limiting simple activities of daily living, such as bathing, dressing and undressing, feeding self, using the toilet, taking medications, and bedridden for much of the day. None of our patients at the onset of fatigue had tumor progression, increased TSH levels or alteration in hemoglobin levels, sodium levels, potassium levels, glycemia, albumin concentration, and abnormal hepatic and renal functions. Furthermore, there were no other known causes of fatigue in our patients. (**a**) Fatigue in PAI patients. All seven patients with PAI had fatigue (see Results). At the time of PAI diagnosis, three patients had grade 3 fatigue (#6, #9, and #10) and four patients had grade 2 fatigue (#4, #8, #11, and #12). After starting replacement CA therapy, six patients (#4, #6, #8, #9, #10, and #11), which constitutes 85.7%, had a significant improvement in fatigue that persisted during follow-up in five patients (#4, #6, #9, #10, and #11), without the need to change lenvatinib dosage. Two patients (#9 and #10) resolved fatigue with AC therapy. (**b**) Fatigue in patients with no-PAI. Four patients (#1, #5, #7, and #13) in the no-PAI group had fatigue. In two (#1 and #5) of the four patients, an improvement in fatigue was observed after reducing lenvatinib dosage: patient #1 reduced lenvatinib for other adverse events and patient #2 reduced lenvatinib due to the severity of fatigue (grade 3). CA, cortisone acetate; CTCAE, Common Terminology Criteria for Adverse Events; TSH, thyrotropin. Color images are available online.

Seven of 11 patients with fatigue developed PAI (63.4%); therefore, the prevalence of fatigue was 100% in the PAI group and 66.7% in the no-PAI group (*p* = 0.1923) (Fig. 3).

The prevalence of severe fatigue (grade 3) was higher in the PAI group, 42.8% (3/7 patients), than in the no-PAI group, 16.7% (1/6 patients) (Fig. 3), but the difference was not statistically significant (*p* = 0.5594).

CA significantly reduced fatigue in 85.7% of all PAI patients (*p* = 0.0152) (Fig. 3a), without changing lenvatinib dosage, also in severe fatigue; on the contrary, in the no-PAI group, a reduction of lenvatinib dosage was necessary in grade 3 fatigue (Fig. 3b).

Fatigue was not associated with initial weight, ECOG status, sex, follow-up duration, or starting dose or dose intensity of lenvatinib.

Age correlated positively with development (*p* = 0.0007, *r* = 0.8124, CI 0.4733–0.9418) and with the severity of fatigue (*p* = 0.0166, *r* = 0.6482, CI 0.1513–0.8836). There was a tendency for a positive association between fatigue severity and PAI (*p* = 0.0527; *r* = 0.5476).

## Discussion

In this study, PAI was observed in 54% of cases, often in the first 12 months of lenvatinib treatment. According to current guidelines, we used a high-dose ACTH test: the “gold standard” for the diagnosis of PAI (5). The association with increased ACTH levels is consistent with the diagnosis of PAI (5).

Our results agree with those of Colombo *et al.*'s study, which demonstrated PAI in 57% of the seven patients treated with lenvatinib (4). However, these authors evaluated adrenal function only during lenvatinib treatment and not before therapy.

The strength of our study was the ability to evaluate adrenal function before lenvatinib treatment, demonstrating normal adrenal function in all cases.

Considering its prevalence, PAI may be defined as one of the most common, recently observed AEs of lenvatinib.

The negative adrenal effect of lenvatinib may be transient, and recovery of adrenal function may be obtained after lenvatinib interruption. In fact, in one of our patients with PAI, the interruption of lenvatinib was associated with a recovery of adrenal function.

PAI is a severe and potentially life-threatening condition, and its early diagnosis is important (5–7). Interestingly, by evaluating the first ACTH-test, before starting lenvatinib treatment, we found, based on our analysis of only 13 patients, that patients with a <646.6 nmol/L cortisol peak had an increased risk of developing PAI during lenvatinib treatment. We suggest that baseline ACTH-testing and ACTH levels in patients selected for lenvatinib therapy might help inform the need for subsequent evaluation for PAI.

An apparent limitation of our study is that we did not evaluate levels of renin and aldosterone. We chose not to evaluate the levels of these hormones because they were altered by antihypertensive treatments. Hypertension was present in eight patients (61.5%) before starting lenvatinib treatment and in all patients during follow-up. However, sodium and potassium levels were always normal, indicating that mineralocorticoid function was preserved.

We did not evaluate levels of salivary cortisol or free urinary cortisol in our study. Plasma total cortisol levels can be affected by potential changes in cortisol-binding globulin. This may be a limitation of the present study. However, as reported, the limitations of the measurement of total cortisol are typically less important in the evaluation of PAI (high ACTH) than in the diagnosis of secondary or tertiary AI (low or inappropriately normal ACTH) (10).

Another potential limitation of our study may be that adrenal autoantibodies were not determined. However, all our patients had normal adrenal function before lenvatinib treatment, and when PAI developed, mineralocorticoid function was preserved, excluding autoimmune adrenalitis (11).

In almost all patients with PAI in our study, ACTH levels were not as high as those observed in the diagnosis of non-lenvatinib-related PAI (usually greater than twofold the upper limit of range) (5) (Fig. 2). This observation may be explained by the early diagnosis of PAI in our patients, according to our study design, or milder PAI associated with lenvatinib treatment, which prevented a significant increase in ACTH levels.

The mechanisms involved in the development of lenvatinib-induced PAI are unknown. Lenvatinib is a multitargeted TKI; in particular, two of its targets, the vascular endothelial growth factor (VEGF) receptors (VEGFRs) and platelet-derived growth factor receptor alpha (PDGFRα), may be implicated in adrenal control.

Anti-VEGF action markedly reduces vascular density in the adrenal cortex in animal studies (12,13). Interestingly, VEGF expression is more marked in the fasciculata than in the zona glomerulosa in the bovine adrenal cortex (13). Therefore, the inhibition of the VEGF pathway by lenvatinib may reduce the production of glucocorticoids.

Fatigue is one of the most common treatment-related AEs. In the real-life studies, fatigue was observed in 39.1–100% of cases (14–21), in agreement with our work (84.6%).

The mean time for the first onset of fatigue in our patients was 6.64 ± 6.6 weeks, in agreement with Haddad *et al.* (6.1 weeks) (22).

The development and severity of fatigue were associated with age; in particular, in our study, age >60 years significantly increased the risk of fatigue during lenvatinib treatment. We found a similar situation for PAI, which was observed only in patients aged >60 years; however, for PAI the different prevalence was not statistically significant. It is known that older patients are more likely to experience AEs from lenvatinib treatment (23).

Fatigue management is a challenge for physicians, and many conditions may be involved, including anemia, stomatitis, decreased appetite, weight loss, diarrhea, nausea, vomiting, electrolyte disturbances, and increased levels of thyrotropin (TSH) (1,24,25). In many cases, as also in our study, a specific condition cannot be identified and lenvatinib must be discontinued, interrupted, or reduced in dosage, with a possible worsening of treatment efficacy (1).

In our study, severe fatigue (grade 3) was observed in 42.8% of patients and in 16.7% of patients without PAI. There was a tendency for a positive association between fatigue severity and PAI, but this did not reach statistical significance, probably due to the small number of cases.

CA improved fatigue in 85.7% of patients with PAI, without having to reduce lenvatinib dosage. In contrast, in patients without PAI, grade 3 fatigue required a lenvatinib dosage reduction. It is important to underline how the advantages of glucocorticoid therapy were obtained with physiological doses of CA (25–37.5 mg/day), according to the current guidelines (5).

The combination of these observations shows that PAI may be one of the many causes of lenvatinib-related fatigue, in agreement with a previous study (4). However, the sample size of this study was small; as a result, the CIs were wide.

In conclusion, our study confirms that lenvatinib induces PAI, and strengthens the evidence by assessing adrenal function both before and during treatment. Moreover, through the first assessment of adrenal function, both before and during treatment, this is the first study that strongly suggests that lenvatinib induces PAI. PAI must be considered one of the most common AEs associated with lenvatinib. Our data also strongly suggest that PAI may be involved in lenvatinib-related fatigue, particularly in patients who experience extreme fatigue. However, further studies are required to confirm these findings. Underdiagnosis and undermanagement of PAI in patients with RR-DTC, both potentially life-threatening conditions, may have a negative impact on the morbidity and mortality of patients treated with lenvatinib. We propose evaluating adrenal function in all patients selected for lenvatinib treatment to potentially inform the risk of developing PAI. The majority of patients on lenvatinib experience fatigue and they should be assessed for PAI.
